# Intermediate English as a Foreign Language learners’ formulaic language speaking proficiency: Where does the teaching of lexical chunks figure?

**DOI:** 10.3389/fpsyg.2022.949675

**Published:** 2022-08-02

**Authors:** Hani Hamad M. Albelihi

**Affiliations:** Department of English and Translation, College of Sciences and Arts, Qassim University, Buraidah, Saudi Arabia

**Keywords:** EFL learners, formulaic language, intermediate, lexical chunks, speaking proficiency, CSR

## Abstract

This research aims to investigate the impact of learning lexical chunks on the English as a Foreign Language (EFL) Saudi learners’ (aged 13 to 17) speaking fluency. The study uses an intervention with intermediate Saudi learners comprising lexical chunks based upon the books *Collocation in Use and Common Idioms in English*. Findings obtained from the post-test show that the experimental groups scored significantly better when compared to their performance in the pre-test of speaking fluency. On the contrary, the difference in the performance of the control group between the pre and post-tests is not significant as far as speaking fluency is concerned. The findings also show that the experimental group participants had favorable sentiments regarding explicit lexical chunk training. The research has theoretical and practical consequences in teaching and learning a foreign/second language.

## Introduction

Today, experts (e.g., Toub et al., 2018; Carpenter et al., 2020; Verzella, 2020) agree on the ubiquity of chunks in language formation; for example, studies (e.g., Nergis, 2021; Indriyani et al., 2022; Khoiriyah and Mujiyanto, 2022; Monica, 2022) on the importance of multi-word chunks in developing communicative ability show that numerous elements participate in making the speech more natural. One of which is the extent to which learners use multi-word chunks, as well as conversation patterns or established idioms. Being native-like is a matter of concern for many English as a Foreign Language (EFL) learners as they strive to attain speaking proficiency and grasp the intricacies of speaking skills. However, EFL students encounter a plethora of barriers that hinder them to communicate effectively, despite spending a significant amount of time and money toward this goal in various institutions, schools, and educational and/or commercial applications. To illustrate this, students may complete the program requirements, but they almost always fail to meet the standards for fluency in speaking.

As per my experience, learners in EFL contexts lack adequate exposure to the English language when they are being outside the institutional settings; therefore, they are frequently unaware of the changes in lexemes (one of the language issues EFL learners confront) between the L1 and the L2. To confront this issue, I looked for reasons for low-level students speaking proficiency and I came across researchers like Bahari (2019). He stated that when performing responsibilities effectively, low-level educators depend on a lexicalized network of interactions that heavily relies on vocabulary and remembered chunks of language. In the same vein, Green and Lambert (2018) claimed that a lexical approach that emphasizes chunks does not ignore the structural aspects of language. Similarly, Al-Ahdal et al. (2014) and Unsworth and Mills (2020) affirmed that we might explain little without grammar, but nothing can be expressed without vocabulary. Moreover, Mumford and Dikilitaş (2020), the founders of the “Lexical Approach,” claim that lexis, or words and word combinations, are the building blocks of learning the language and communication, not the function, terms, syntax, or any other unit of teaching and planning. Native speakers, according to Verzella (2020), have a repertory of various routines of word blocks, or “chunks.” They must be mastered and utilized as fixed terms in suitable settings to develop a conversation that sounds natural and native-like.

Conrad and Biber (2005) theoretical model was the most prevalent used in the classification of formulaic expression. However, this study did not aim to classify such expressions. It instead focuses on the impact of teaching lexical chunks on developing students’ fluency. Hence, several previous studies explored the impact of EFL lexical learning pertained experimental design to gauge the effectiveness of interventions (e.g., Al-Gahtani and Roever, 2018; Kidd et al., 2018; Taguchi, 2018; Tsai and Tsai, 2018; Lim, 2019; Rosenhan and Galloway, 2019; Shirazizadeh and Amirfazlian, 2021; Abdalhussein, 2022; Khoiriyah and Mujiyanto, 2022). Therefore, this study will obtain such a design.

### Significance of the study

The importance of this study springs from the assumption that it will eventually offer scientific justification for the hypothesis that lexical chunks training can enhance EFL students’ speaking fluency through reading-speaking communication programs. thus, helping them conquer several of the challenges when their ability to speak fluently is highlighted. As a speaking skilled teacher, the researcher’s experience has shown that EFL students at English institutions, particularly teens, face many obstacles in speaking. This problem stemmed from the fact that most of them have never spoken English in school. Most struggle to organize their thoughts, use proper organization, apply English speaking techniques, and locate appropriate words to explain their views.

### Literature review

Little attention has been paid to lexical chunks in most conventional work on formal and theoretical linguistics. Most lexical chunks advocates come from language studies and instruction, notably foreign language education (Omidian and Siyanova-Chanturia, 2021; Shirazizadeh and Amirfazlian, 2021). One of the reasons for the paucity of consideration of lexical chunks in formal languages is that it is a challenging phenomenon to nail down formally. Because lexical chunks incorporate semantic, syntactic, and even pragmatic information, they do not fit neatly into established linguistic categories. However, lexical chunks are seldom discussed in formal linguistics because they are ambiguous in clear, universal terms. Notwithstanding this, efforts were made, and the researcher will discuss some of the most prominent research linked to the current topic.

Previous research is available on the effects of lexical chunks on writing. Omidian and Siyanova-Chanturia (2021) conducted empirical research on using lexical chunks on writing skills. The study focuses on whether using a lexical chunk approach in EFL education can assist college students in enhancing their EFL writing skills. The findings of the study demonstrated that the lexical chunk teaching/learning technique helps college students improve their English writing.

Similarly, Shirazizadeh and Amirfazlian (2021) explored the effects of lexical bundles on the fluency of Saudi EFL learners in producing paragraphs. They administered an English language competency exam to 120 EFL students, and based on their proficiency test results, they chose ninety learners and separated them into experimental and control groups. Their investigation revealed that lexical bundles training had a substantial impact on the experimental group learners, and in the absence of any other change, this was attributed to the lexical bundles’ education they received.

The idea behind chunk studies is that native speakers employ many chunks in their everyday conversation, which identifies them as proficient speakers of the language (Gilmore and Millar, 2018; Carrol and Conklin, 2020). Saeedakhtar et al. (2020) results on the significance of multi-word strings on the concept of fluency showed that mastering ready-made chunks might assist students in improving their English fluency. Participants were informed about the benefits of utilizing chunks in their speech and were urged to do so throughout the semester.

Enayat and Derakhshan (2021) looked at the impact of teaching collocations on the speaking skill of Saudi EFL students and the link between the respondents’ understanding of collocations and their usage of collocations. They also tried to determine how the participants felt about being taught collocations. Their findings showed the effectiveness of teaching collocation to increase collocation understanding. They went on to say that after learning the collocations, students will be able to have better control over their English-speaking abilities and grasp the themes in dialogs and discussions.

In recent years, researchers have shown an academic interest in chunk learning. Several studies were conducted in different settings. They all reported the fundamental role formulaic expressions play in enhancing EFL learners’ fluency in the target language. Abdalhussein (2022) explored the differences between high and low Iraqi EFL students using formulaic expressions in test writing proficiency. The study pertained discourse makers model and mixed data collection and analysis methods. Findings showed that some interactive and meta-discourse markers were more prevalent than others. Furthermore, the study reported that higher proficiency students used formulaic expressions more than low proficiency students. The study recommended the importance of activating salient discourse makers by both EFL teachers and curricula writers.

Furthermore, Monica (2022) gauged the effects of using multiword groups in boosting EFL students’ communicative ability. The study obtained two groups designed. The experimental groups were exposed to the practice of chucking, while the control group was taught traditionally. At the end of the course, a post (speaking) test was given to both groups. The finding showed that the experimental group used chucking more significantly than the control group. Moreover, the experimental group developed positive attitudes toward the practice of chunking for developing their speaking ability.

Studies obtained Conrad and Biber (2005) theoretical model while analyzing the gathered data. Khoiriyah and Mujiyanto (2022) explored the formulaic expressions of Kampung Inggris Pare students. The study recorded students’ conversations through observation to analyze their performance in using chunking. The study reported that the participants uttered several types of formulaic expressions. They made some collocational problems like combining two particles. Students simple multiword combinations and avoid more complex ones. Furthermore, high proficiency students produced more formulaic expressions. Likely, Indriyani et al. (2022) investigated the Semarang EFL teachers and students use five types of chunks in their classroom communication. The study obtained thirty-three participants as the sample (two teachers and thirty-one students). The conversations were analyzed. The study reported that all five types were perceived: idiomatic phrases, lexical bundles, binomial expressions, free verb + particle combinations, and inserts. The study reported that no significant difference was found between teachers and students in using the formulaic expression. It was found that students used some expressions that the teachers did not, in any case.

Furthermore, still, other studies investigated the impact of the pedagogical approach in developing EFL formulaic expressions. Cancino and Iturrieta (2022) reported the importance of exposing students to natural chucking to develop their fluency. The study detects the impact of using a lexical approach on developing EFL formulaic expression and oral proficiency. The study recruited two groups. The first was exposed to thirty-eight teaching hours using a lexical approach, while the other was taught traditionally. Both groups appeared in a speaking task. Findings revealed that the experimental group scored higher in the proficiency tests and the number of used formulaic expressions. Furthermore, the study showed a correlation between oral proficiency and the use of formulaic expressions. In the same vein, Nergis (2021) compared the effectiveness of two pedagogies on EFL fluency. The first group learns through formulaic sequences in spoken class, while the other learns chunks through academic vocabulary sessions. The interventions lasted after 5 weeks. An oral test based on three evaluation aspects (complexity, accuracy, and fluency) was given to both groups. Findings revealed that the first formulaic sequence group scored higher than the academic vocabulary group in fluency, while the academic vocabulary group outperformed the first group in complexity.

In the same vein, researchers like Toub et al. (2018), Carpenter et al. (2020), and Verzella (2020) confirmed that chunks and formulaic units of language help EFL learners in boosting their fluency by forming sentences and lengthening their speech. These researchers hypothesized that by memorizing many packages and retrieving them spontaneously, students might achieve fluency with native-like speakers and improve the duration of pauses between speech. Speaking fluency in this research is defined according to Liao et al. (2018) as the number of words in a T-unit, regardless of whether they are repeated. Word, caprolactone, prepositional phrases, sentence patterns, moderate gestures, compounding, grammatical patterns, idiomatic, constant phrases, and prefabricated are the ten categories of lexical chunks.

### Research questions

A review of the available literature on the role of instruction in lexical chunks has led the current research to answer the following questions in the Saudi EFL learners’ context:

1. Does lexical chunks education affect the fluency of Saudi EFL learners while speaking?

2. Is there a substantial distinction in scores between the experimental and control groups’ performance attributable to the intervention?

## Materials and methods

### Research design

The study applied an experimental design to detect the impact of lexical learning on students’ fluency. The group is then divided equally into experimental and control groups. As a pre-test, the learners are given a ten-question interview. Following this, an intervention.

### Respondents

The study participants were intermediate-level Saudi male learners aged 12 to 17, with a median age of 15 years. Before entering the academy, the bulk of them attended public schools. They were all learners with an intermediate level of non-native English studying a coursebook called Headway 3. Due to their restrictions, the English institutes could not devote any time to research. Therefore, the intermediate respondents of this English teaching institution were chosen using the accessibility sampling technique in this study. The investigator used a 60-question Quick Oxford Placement Test (QOP) to homogenize the individuals and pick the intermediate-level respondents. Following this, sixty participants were selected randomly from a pool of 120 after subjecting them to the Quick Placement Test (QPT). Stratified random sampling was obtained to homogenize the participants in each group. The researcher depended on the students’ QPT to group the participants into three strata (high, medium, and low) and select twenty students to represent the high-level students, twenty for medal level students, and twenty for low-level students. Hence, the application of random strata assures the representativeness of the population and allows for the generalization of the findings. The chosen students were split into two groups of thirty students (10 from each stratum) each (as stated earlier, into experimental and control groups). The groups followed the following:

The research adopted a two-group design (control and experimental) to accomplish this result. Thus, to address the research problem, the participants were split into two groups (experimental and control) randomly using TOEFL to measure their pre-intervention English skills. However, treatment in the form of focused instruction in lexical chunks was administered to the experimental class alone. During the treatment, as part of the activities, the intervention class used lexical chunks to create many cohesive paragraphs on various themes; these were subsequently delivered and debated in class. They were expected to employ lexical chunks in their interactions while the researcher observed them. Lastly, they take roles in the class dialogs.

Meanwhile, the control group students did not get specialized lexical chunk exposure. They just got a simulated treatment. They followed the same textbook (Headway) using the traditional language education approach of grammar-translation with no lexical chunk training. The variables considered in the study were learning lexical chunks (independent variable) and speaking fluency as the two factors central to this research (dependent variable).

### Instrumentation

The Quick Proficiency Exam (QPT), a collocation test, and an interview were used in this research. The first instrument utilized in this research was the QPT version 1, which consisted of two components. It was used to detect the homogeneity of the respondents and determine their proficiency level. There were forty questions in the first portion and twenty questions in the second. To participate in the research, volunteers needed to have a score that was one measure of the spread above or below the mean.

The second part of the test was a lexical chunks test, which served as both a pre- and post-test. It consisted of thirty multiple choice questions given to all sixty respondents, with scores ranging from 0 to 30. The lexical pieces were chosen from Malyuga and McCarthy’s (2018) “English Collocations in Use.” They were chosen based on the book (18 units) in question. The reliability of the lexical chunks test was previously computed and found to be 0.82, which is appropriate for this kind of investigation.

The third assessment criterion was an open-ended interview of ten-minutes duration that served as both a pre-test and a post-test. It comprised ten questions that allowed the learners to express their significant thoughts and ideas while supporting them with the chunks they had studied over the eighteen study sessions. The interviews were conducted with the experimental group of students, spanning 10 min per interview. These were recorded with consent, transcribed, and later analyzed to crystallize students’ reactions to the use of instruction in lexical chunks in English.

The exam consisted mainly of a production component in which respondents were required to speak on about ten different themes using the studied lexical chunks. The interview’s inter-rater dependability was evaluated and found substantial for the research.

### Procedures

In each session, the participants were given a lesson on Collocations in Use and ten lexical chunks mostly fixed phrases and idioms. The experimental group participants were instructed to highlight the lexical chunks and apply them in their sentences to learn the lexical chunks. The teacher demonstrated to the class the various methods by which lexical chunks can be fitted into common parlance. Each lesson in the book (Malyuga and McCarthy, 2018) provides two-word lexical chunks, mostly adjective-noun, verb phrases combinations, and the most popular idioms that the subjects may use to increase the number of words in their T-units. Each session, the learners were given a unit and then encouraged to do the lexical chunks tasks by creating sentences using those chunks. They were also required to complete the activities of the same unit on the following pages. They were instructed to skim the lessons, highlight the idioms and collocations, and use them in sentences of their own once they had been taught each subject. The teacher would go over the new unit in the following sessions. The teacher followed the method of asking questions in earlier classes as well. The teacher would choose the units for instruction based on their value, and units with more valuable lexical chunks in spoken English were often picked. The individuals in the control group, on the other hand, were only requested to examine the course necessary sections, Headway Book 3, and were not given any additional or extracurricular assignments.

After 2 weeks, post-tests were administered to both the groups in speaking fluency that included an interview on the same themes as the pre-test and a collocations exam comparable to the pre-test but included different collocations and lexical chunks. The respondents were asked to report their thoughts on ten major topics they may confront in their lives as part of the interview process. The researcher concentrated on the amount of lexicon in the T-units when analyzing the respondents’ post-test transcriptions to see whether they had improved their speaking fluency, which contains several example assignments. The conversations were recorded, transcribed, and analyzed to see whether there were any great disparities in speaking fluency between the two groups. Aside from some comforting queries, the interviewer had ten essential questions to ask. The number of words per t-units (W/T-unit) to assess speaking fluency by Bice and Kroll (2019). SPSS statistical software was used to analyze the raw scores once they were measured. The *t*-test was used to calculate the statistical differences in the means of the scores in both tests in the two groups. A statistician analyzed the raw data using SPSS statistical software to achieve this goal. It was also chosen to look at the ten different sorts of lexical chunks described in Gilmore and Millar (2018) and Liao et al. (2018), which are given.

## Results

This section takes each of the research questions systematically and uses the data to obtain answers to these.

### RQ1

Does lexical chunks education affect the fluency of Saudi EFL learners while speaking?

The first question was answered by showing the students’ fluency scores in the speaking test. Table 1 shows that students in the pre-test scored 9.981 with a standard deviation of 1.4950. After receiving some treatment, the same group scored in the post-speaking test 12.021 with a standard deviation of 1.3752. According to Table 1, students displayed some improvement in speaking fluency with a mean score of (2.04). The difference between students’ scores in the pre-speaking fluency test and post-speaking fluency test is significant, as the sig (two-tailed) value showed 0.000.

**TABLE 1:** Experiment group speaking fluency.

Paired samples statistics					*T*-test		
Pair one	*N*	Mean	Std deviation	Range	*t*	df	Sig (two-tailed)
Pre-test	30	9.981	1.4950	2.04	−7.7219	2	0.000
Post-test	30	12.021	1.3752				

Experimental group students reported some attitudes regarding chunk learning. The interview amazed four dominant themes in the responses were cantered around (i) ease of use in speaking with a remote repository of formulaic language; (ii) boosted confidence in speaking in English in front of peers; and (iii) opinion that their course materials should focus on giving them ready-to-use expressions in speaking in English.

To triangulate the data from the test, three participants added that they would rather have similar formulaic chunks given to them for better English writing skills. One participant reported that with the exposure to lexical chunks used in English, he felt more confident in his other language skills, writing.

### RQ2

Is there a substantial distinction in scores between the experimental and control groups’ performance attributable to the intervention?

To answer the second question, a *t*-test was used to compare the groups’ (control and experimental) principal scores in the post-test. Table 2 shows that the control group scored (10.05) in the speaking fluency post-test with and standard deviation of (1.9686) whereas the experimental group scored (12.021) in the post-speaking fluency test with and standard deviation of (1.3752). Despite the slight difference that the experimental group students achieved in the post-test compared to their control group counterparts, the difference seems insignificant because the sig (two-tailed) value is 0.3971, which is more significant than 0.05.

**TABLE 2:** Post-test scores of controls and experimental groups.

Categorized of statics					*t*-test	
	Post-test	*N*	Mean	Std deviation	*t*	Sig (two-tailed)
Fluency scores	Control	30	10.05	1.9686	1.9733	0.3971
	Experiments	30	12.021	1.3752		

This numerical finding can be triangulated by the interview results, as eleven of the respondents in the experimental group complained that their EFL learning had not made them as confident in speaking English as the limited instruction in lexical chunks.

## Discussion

Two queries lie at the core that the study at hand tried to answer. Regarding the first query, the study found that training on lexical chunks plays a significant role in developing students’ fluency in speaking. The study compared the scores of the experimental group itself in the pre and post-test and found a significant difference between them, as there is an increase in the mean score of the experimental group in the post-test. In other words, the experimental group students scored higher in the post-test. This result is a bit ambiguous, as no one can say the improvement is a result of the treatment that the students’ received because there were many variables that no one can control. One of them is the teaching methodology of the coursebook that they studied. This finding is in line with (Toub et al., 2018; Bahari, 2019; Al-Ahdal and Alkhalaf, 2020; Carpenter et al., 2020; Verzella, 2020; Enayat and Derakhshan, 2021; Abdalhussein, 2022; Cancino and Iturrieta, 2022; Khoiriyah and Mujiyanto, 2022). Enayat and Derakhshan (2021) showed that teaching lexical collocations is valuable for maximizing approximation knowledge. As an outcome, EFL learners can manage to have more control over their speaking ability in English. Furthermore, the findings of this research support Toub et al. (2018), Carpenter et al. (2020), Verzella (2020), Indriyani et al. (2022), and Monica (2022), which found that lexical bundles and formulaic language units aid second language learners in boosting their speech speed by allowing them to construct longer sentences.

Furthermore, the study reported that the experimental group outperformed the control group in the post-test regarding the second query. However, the differences between the experimental and control mean scores were not significant. The interview results confirm this finding. Most of the interview students reported that lexical learning could not boost them to become confident in language use. Likely, this finding confirmed the researcher’s hesitation when he presented the first research question above. It meant that the differences between the experimental group scores in the pre and post-test need not only relate to the treatment they received on chunk learning. This finding needs more exploration and study in further research.

## Conclusion

The current research was concerned with how EFL students may improve their speaking fluency by using the reading-into-speaking approach and exposing them to extracurricular lexical pieces. It investigated differences in students’ speaking overall performance of fluidity, which is one of the indicators of successful speaking. The findings imply that exposure to vast lexical chunks of reading and learning aided the development of speaking fluency in these intermediate or lower intermediate level EFL students.

Other conclusions drawn from the research show that the experimental group’s speaking fluency was greater than the control group. One potential explanation is that the students had more opportunities to read and work with lexical chunks in varied texts. Thus, in the experimental group, learning chunks played a considerable influence on the speaking fluency of the participants. However, the slightly higher scores that the experimental group got when compared with the control group, also implied that the difference between them was not significant. So, the achievement in the post-test scores of the experimental group may relate to another variable that the researcher could not control during the study.

### Recommendations

The study reached some pertinent conclusions based on the data, which also helped the researcher make specific recommendations. This research establishes the role of instruction in achieving higher speaking fluency. This finding recommends that communicative proficiency rather than core language knowledge should be the focus of EFL teachers, especially with intermediate or lower-level learners. Such formulaic language training boosts the learners’ morale, motivates them to strive for better fluency, and removes the cognitive overload when forced to work from scratch. Similarly, students’ fluency may be measured by the ability to use and comprehend the most frequent words in English. It is thus recommended that both teachers and students should be familiarized with the use of corpora, i.e., The American National Corpus (ANC), the Corpus of Contemporary American English (COCA), and the British National Corpus (BNC), where student can check their language use. Further, textbooks and other classroom materials should be revised to suit the needs of the learners, of which communicative fluency is undoubtedly a leading one.

Chunk learning plays a key role in second language acquisitions; however, research in this field needs more sophisticated and innovative ways of analyzing the findings. More future studies are needed to analyze the role of the environment in developing the students’ chunks of acquisitions. By environment, the researcher means the social media and other shared platforms that may play an active role in developing the levels of students in such types of lexemes and which may participate in the students’ acquisition level.

### Limitations

Though a pathbreaking study in the direction it has taken, this study also has certain limitations. The negation of the variable of gender has been one of these, which makes the applicability of the results doubtful in more significant and varied environments. In addition, the researcher has admitted that the change in the performance may be attributable to some other factors that were not controlled by the study plan. Similarly, speaking fluency or performance should be assessed in a real-life situation. Many students who speak confidently in the classroom (because they are aware of the topics being discussed) may not perform the same in contexts where the aim of the conversation is interpersonal. This makes it pertinent for future studies to broaden the study variables and evaluate students’ competence in real-life contexts with native speakers, for example. Finally, more extensive control and experimental groups will also lend excellent reliability to the findings, a rejoinder for future work in this direction.

## Data availability statement

The original contributions presented in this study are included in the article/supplementary material, further inquiries can be directed to the corresponding author.

## Author contributions

The author confirms being the sole contributor of this work and has approved it for publication.
