# Corrigendum: Intermediate English as a Foreign Language learners' formulaic language speaking proficiency: Where does the teaching of lexical chunks figure?

**DOI:** 10.3389/fpsyg.2022.1031974

**Published:** 2022-10-20

**Authors:** Hani Hamad M. Albelihi

**Affiliations:** Department of English and Translation, College of Sciences and Arts, Qassim University, Buraidah, Saudi Arabia

**Keywords:** EFL learners, formulaic language, intermediate, lexical chunks, speaking proficiency, CSR

In the published article, there was an error in the affiliation. Instead of “Department of English Language, College of Arts and Sciences, Methnab, Qassim University, Buraydah, Saudi Arabia” it should be “Department of English and Translation, College of Sciences and Arts, Qassim University, Buraidah, Saudi Arabia”.

The author apologizes for this error and states that this does not change the scientific conclusions of the article in any way. The original article has been updated.

## Publisher's note

All claims expressed in this article are solely those of the authors and do not necessarily represent those of their affiliated organizations, or those of the publisher, the editors and the reviewers. Any product that may be evaluated in this article, or claim that may be made by its manufacturer, is not guaranteed or endorsed by the publisher.

